# Activity dependent modulation of glial gap junction coupling in the thalamus

**DOI:** 10.1016/j.isci.2024.111043

**Published:** 2024-09-26

**Authors:** Paula Baum, Anna Beinhauer, Lara Zirwes, Linda Loenneker, Ronald Jabs, Rajeevan T. Narayanan, Marcel Oberlaender, Gerald Seifert, Helmut Kettenmann, Christian Steinhäuser

**Affiliations:** 1 Institute of Cellular Neurosciences, Medical Faculty, University of Bonn, 53127 Bonn, Germany; 2 In Silico Brain Sciences Group, Max Planck Institute for Neurobiology of Behavior - caesar, 53175 Bonn, Germany; 3 Department of Integrative Neurophysiology, Center for Neurogenomics and Cognitive Research, VU Amsterdam, 081 Amsterdam, the Netherlands; 4 Cellular Neurosciences, Max-Delbrück-Center for Molecular Medicine in the Helmholtz Association, 13125 Berlin, Germany; 5 Shenzhen Institute of Advanced Technology, Chinese Academy of Sciences, Shenzhen, China

**Keywords:** molecular neuroscience, cellular neuroscience

## Abstract

Astrocytes and oligodendrocytes in the ventrobasal thalamus are electrically coupled through gap junctions. We have previously shown that these cells form large panglial networks, which have a key role in the transfer of energy substrates to postsynapses for sustaining neuronal activity. Here, we show that the efficiency of these transfer networks is regulated by synaptic activity: preventing the generation and propagation of action potentials resulted in reduced glial coupling. Systematic analyses of mice deficient for individual connexin isoforms revealed that oligodendroglial Cx32 and Cx47 are the targets of this modulation. Importantly, we show that during a critical time window, sensory deprivation through whisker trimming reduces the efficiency of the glial transfer networks also *in vivo*. Together with our previous results the current findings indicate that neuronal activity and provision of energy metabolites through panglial coupling are interdependent events regulated in a bidirectional manner.

## Highlights

• In the thalamus, panglial gap junction coupling is regulated by synaptic activity
• In juvenile mice, sensory deprivation *in vivo* reduces coupling efficiency
• Oligodendroglial Cx32 and Cx47 are the targets of this modulation
• Neuronal activity and coupling-mediated metabolite transfer influence each other

## Graphical abstract

**Figure:**
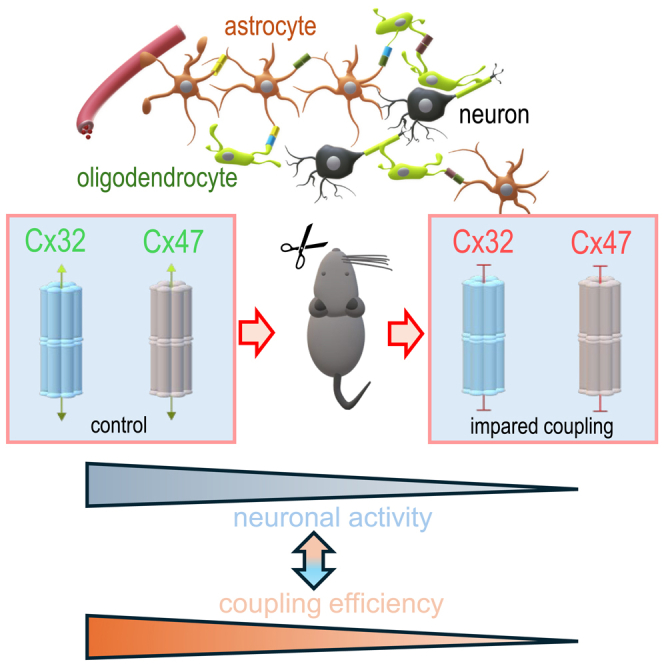

## Introduction

The thalamus is involved in controlling and passing on information to the neocortex. The ventral posterior nucleus (VP), operating as a somatosensory relay, contains elongated structures called barreloids, representing the somatotopic vibrissae organization.^1^^,^^2^ Part of the VP is the ventroposteromedial nucleus (VPM), which receives input from the contralateral vibrissae of the whisker pad^3^ and projects to the primary somatosensory neocortex.^4^^,^^5^^,^^6^ Astrocytes and oligodendrocytes in the thalamus are abundantly coupled through gap junctions, constituting large panglial networks, while panglial coupling is much less prevalent in the neocortex and hippocampus.^7^ Gap junction channels are formed by different connexin (Cx) isoforms and the expression pattern varies with cell type, brain region, and developmental stage.^8^^,^^9^^,^^10^ In the neocortex and hippocampus, functional coupling of astrocytes mainly depends on Cx43 (gene name, *Gja1*) and to a minor extent on Cx30 (*Gjb6*). In the thalamus, in contrast, most astrocytes express Cx30 and many even lack Cx43.^7^ Oligodendrocyte coupling relies on Cx47 (*Gjc2*) or Cx32 (*Gjb1*). Panglial networks require heterotypic gap junction channels made by Cx30:Cx32, Cx47:Cx43, or Cx47:Cx30 while Cx43 and Cx32 do not form functional channels.^11^^,^^12^^,^^13^^,^^14^ In the thalamus, panglial coupling is mainly mediated by Cx30:Cx32 channels.^7^ Cx30 levels increase after the second postnatal week^15^^,^^16^ although in the thalamus, Cx30 and Cx43 protein and functional coupling are already present earlier, with coupling efficiency remaining constant from p13 onward.^7^ The borders of barreloids in the VPM are formed by weakly coupled oligodendrocytes, which prevent expansion of coupling networks across multiple barreloid fields.^17^

A common approach to evaluate sensory input into the rodent thalamus is manipulation of the whiskers, e.g., by trimming of vibrissae. The long macrovibrissae of the mystical pad provide spatial information, e.g., by distance detection, and are represented in the barreloids of the VPM. The shorter microvibrissae are important for object recognition.^1^^,^^18^ In mice, active whisking with exploration of the environment begins at p10/11 while object exploration is first observed at around p13, when eyes open.^19^^,^^20^^,^^21^ Whisker trimming leads to reversible changes of thalamocortical axons throughout postnatal life.^22^^,^^23^ Much less is known about consequences of sensory deprivation on thalamic networks,^24^^,^^25^ and neuron-glia interactions in particular.

Glial coupling plays an important role in supplying energy metabolites for maintenance of neuronal activity.^26^^,^^27^^,^^28^ In this study, we now address the question whether neuronal activity could influence the efficiency of panglial coupling and hence the transfer of energy substrates. Such neuronal impact on glial coupling has indeed been shown in olfactory glomeruli where Cx30-based astrocytic gap junctions are sensitive to action potential firing and changes in extracellular K^+^ levels.^29^ Similarly, inhibition of action potentials and presynaptic transmitter release in the thalamus resulted in reduced spread of biocytin and the fluorescent glucose derivative, 2-(*N*-(7-Nitrobenz-2-Oxa-1,3-Diazol-4-yl)Amino)-2-Desoxyglucose (2-NBDG) within the glial network.^17^ Here, we asked which connexin isoform(s) mediate the activity-dependence of panglial coupling in thalamic barreloids and whether these glial transfer networks are controlled by sensory input *in vivo*.

## Results

### Postnatal regulation of connexin transcript expression in the thalamus

Little is known about the expression of connexin transcripts during postnatal development of the thalamus. We performed semiquantitative reverse transcription polymerase chain reaction (sqRT-PCR) to systematically compare the mRNA levels for astrocytic (Cx30 and Cx43) and oligodendroglial connexins (Cx32 and Cx47) between p5 and p45 in the ventrobasal thalamus (for primers and probes, see Table 1). Between p5 and p11, expression of Cx30, Cx32, and Cx47 strongly increased and then remained constant (Figures S1A–S1C, and S1D) while Cx43 transcripts reached adult level already at p5, the earliest time point investigated here (Figure S1B). Our data thus indicate that in the 11-day-old thalamus, all four connexin isoforms are present at quasi-adult level, at least on the transcript level.

**Table 1:** Primers, probes, and assays used for RT-qPCR

Gene	Gene expression assay number Primer and probe sequence	Product length	Position	GenBank accession number
Cx30 (Gjb6)	Mm00433661_s1	67 bp	910–976	NM_001010937.3
Cx32 (Gjb1)	Mm01950058_s1	65 bp	434–498	AK 134742.1
Cx43 (Gja1)	Mm01179639_S1	168 bp	2853–3020	NM_010288.3
Cx47 (Gjc2)	Mm00519131_s1	87 bp	502–588	NM_080454.4
β-actin (ACTB)	Mm00607939_s1	115 bp	1140–1254	BC138611.1
Primers used for developmental expression study				
Cx30 (Gjb6)	se 5′-CGTACACCAGCAGCATTTTCTT as 5′-ACCCATTGTAGAGGAAGTAG AACACAT probe FAM-5′-CGCATCA TCTTCGAAGCCGCCT-TAMRA	78 bp	705–726 756–783 728–754	NM_001010937.3
Cx43 (Gja1)	se 5′-TTTGACTTCAGCCTCCAAGGA as 5′-TCTGGGCACCTCTCTTTCACTTA probe FAM-5′-TTCCACCACTTTGGC GTGCCG-TAMRA	79 bp	153–173 209–231 175–195	NM_010288.3
Cx32, Cx47, β-actin	As above	–	–	–

Length of PCR products is indicated as base pairs (bp). “Se” and “as” mark sense and antisense primers. All sense and antisense primers are located on different exons, respectively. Cx TaqMan probes were labeled with 6-carboxyfluorescein; 5′-end (FAM), 6-carboxytetramethylrhodamine; 3′-end (TAMRA), or a non-fluorescent minor groove binder at the 3′-end.

### Oligodendroglial gap junction coupling depends on neuronal activity

**Figure 1:**
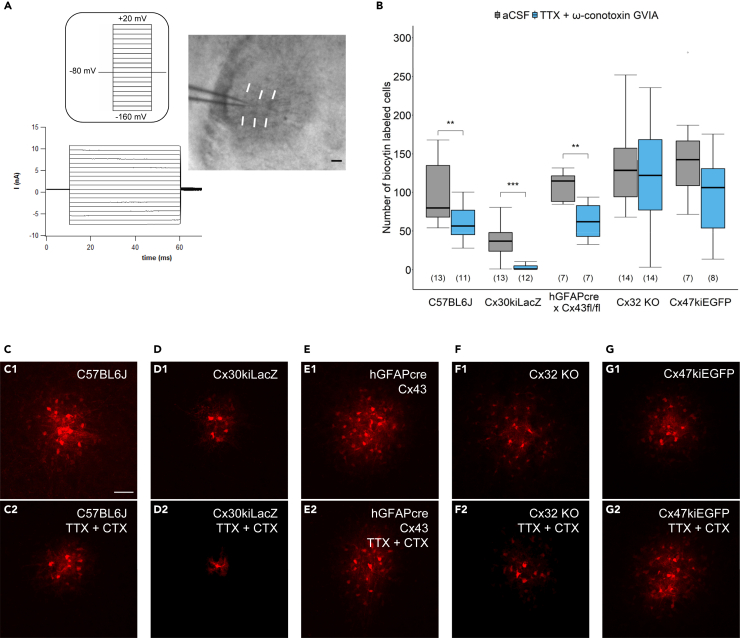
Activity dependent modulation of gap junction coupling in the thalamus (A) A slice containing barreloids was prepared from a C57BL6/J mouse brain (bright field; right). An astrocyte within the barreloid field was approached by the patch pipette. White lines indicate barreloid borders. Scale bar: 100 μm. De- and hyperpolarizing voltage steps (from −160 mV to +20 mV, 10 mV increments, duration 50 ms; inset) evoked a membrane current pattern typical of astrocytes (left). Holding potential was −80 mV. (B) Boxplots indicating the number of biocytin-positive cells as a measure of coupling efficiency under control condition (aCSF) and after pre-incubation with aCSF or aCSF with TTX (0.5 μM) + ω-conotoxin GVIA (CTX) (0.5 μM) for 3–4 h. Inhibition of neuronal activity decreased the spread of biocytin in C57BL6/J mice, Cx30kiLacZ mice and hGFAPcre x Cx43 fl/fl mice. Number of mice is given in brackets. Boxplots represent median and quartiles. Two sample t-test (hGFAPcre x Cx43 fl/fl, Cx32 KO, Cx47kiEGFP mice, log-transformed data for C57BL6/J mice) or Wilcoxon rank-sum test (Cx30kiLaZ mice). Stars indicate statistical significance: ∗∗*p* < 0.01, ∗∗∗*p* < 0.001. (C–G) Representative maximum intensity projections (MIPs) of biocytin-filled astrocytes in the barreloids of the VPM in different mouse lines in aCSF (upper row) and aCSF supplemented with TTX + CTX (lower row). Images depict representative brightness and contrast adjusted regions of interest (ROI). Scale bar: 50 μm.

Thalamic astrocytes and oligodendrocytes form large panglial gap junction networks, which are predominantly mediated by heterotypic Cx30:Cx32 channels.^7^ Coupling efficiency in the barreloids depends on neuronal activity^17^ although the underlying mechanisms remain unclear. We asked which of the glial connexin channels in these structures are sensitive to neuronal activity. Therefore, thalamic slices from wild type (C57BL6/J) and connexin-deficient mice were incubated in tetrodotoxin (TTX) to block action potentials (0.5 μM) and ω-conotoxin GVIA (CTX) to reduce transmitter release (0.5 μM; 3–4 h) prior to filling an SR101-positve astrocyte within the barreloid with biocytin (0.5%) and Texas Red Dextran (0.1%) for 20 min through the patch clamp pipette (Figure 1). To allow visualization of barreloid structures in acute slices, the experiments were performed between p14 and p17. In accordance with previous reports,^17^ neuronal inhibition reduced coupling in slices from wild-type mice (Table 2; Figures 1B and 1C). In mice lacking astrocytic connexins (i.e., Cx30kiLacZ and hGFAPcre x Cx43 fl/fl mice), the coupling networks retained their sensitivity to neuronal activity (Table 2; Figures 1B, 1D, and 1E). Some astrocytes in barreloids from Cx30kiLacZ mice were even completely uncoupled after deprivation of neuronal activity (*n* = 9, *N* = 7; Figure 1D2). In contrast, after deletion of oligodendroglial connexins gap junction coupling in the barreloids was no longer sensitive to neuronal activity. Thus, in Cx32 KO mice dye coupling was not affected by TTX and CTX treatment and, similarly, coupling in Cx47kiEGFP mice was also not affected by deprivation of neuronal activity (Table 2; Figures 1B, 1F, 1G). These experiments suggest that within the thalamic panglial networks, the efficiency of oligodendroglial gap junction coupling is dependent on the activity neighboring neurons.

**Table 2:** Number of biocytin-positive cells in barreloids under control conditions and after application of 0.5 μM TTX +0.5 μM CTX

Mouse line	ACSF	ACSF +TTX + CTX	*p* value
C57BL6	79.8 (68.0–134.5) *n* = 22, *N* = 13	56.5 (45.5–76.8) *n* = 15, *N* = 11	0.008 two-sample t test on log-transformed data
Cx30kiLacZ	37.0 (24.0–48.0) *n* = 17, *N* = 13	1.0 (1.0–5.1) *n* = 18, *N* = 12	0.0003 Wilcoxon rank-sum test
hGFAPcre x Cx43 fl/fl	114.5 (88.1–121.3) *n* = 11, *N* = 7	62.0 (43.1–82.8) *n* = 13, *N* = 7	0.003 two-sample t test
Cx32 KO	128.3 (94.1–157.3) *n* = 21, *N* = 14	122.3 (77.3–168.4) *n* = 16, *N* = 14	0.479 two-sample t test
Cx47kiEGFP	142.5 (108.9–166.5) *n* = 12, *N* = 7	106.4 (54.1–130.7) *n* = 13, *N* = 8	0.125 two-sample t test

Data are given as median and interquartile range (quartile 25% - quartile 75%), *n* = cell numbers, *N* = number of animals.

In comparison to panglial coupling in barreloids of C57BL6/J mice the number of coupled cells in Cx30 knockout mice under control conditions was reduced, because Cx30 is the main astrocytic connexin in the thalamus and dominate number of cell contacts by gap junctions to neighboring astrocytes and oligodendrocytes.^7^ In contrast, coupling in the other connexin KO vs. C57BL6/J mice was similar under control conditions.

### CC1 as a marker for oligodendrocytes in the thalamus

**Figure 2:**
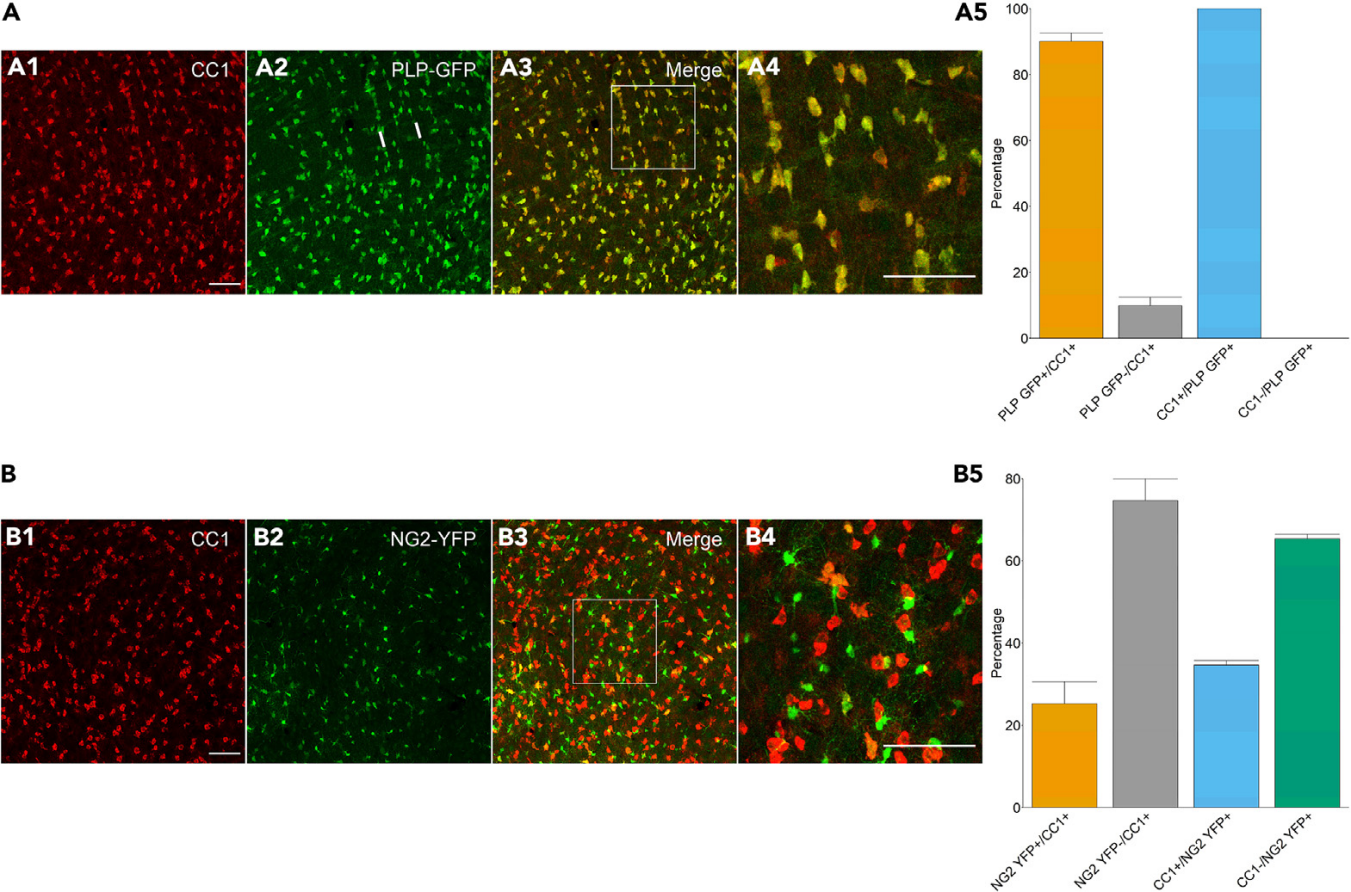
CC1 as a marker for oligodendrocytes in the thalamus (A) Co-staining against CC1 and GFP was performed in PLP-GFP mice at p15/16 (A1–A4). Representative MIPs of combined CC1 and GFP staining in the VPM. Quantitative analysis revealed abundant co-localization of CC1 and PLP-GFP (*n* = 13, *N* = 4; Figure A5). Note the aligned pattern of some PLP-GFP^+^ cells indicating barreloid borders (white bars; A2). (A4) Blowup of boxed area in (A3). (B) CC1 immunostaining was performed in NG2-YFP mice at p15 (B1-B4). Only a minority of cells showed co-localization (*n* = 7; *N* = 3; Figure B5). (B4) Blowup of boxed area in (B3). Bar graphs represent mean ± SD. Scale bars: 75 μm.

Thalamic glial cells express unusual antigen profiles with overlapping expression of common markers for astrocytes and oligodendrocytes, for example glutamine synthetase, Aldh1L1, and Olig2.^7^ To address the question whether the proportion of astrocytes and oligodendrocytes in the coupled networks changes if neuronal activity is suppressed we tested the antibody CC1, which labels mature oligodendrocytes in other brain regions by binding to the RNA-binding protein Quaking 7 (QKI7). QKI7 is highly expressed in oligodendrocytes already at p14 and further increases during maturation and myelin formation.^30^^,^^31^ In the thalamus of mice where the PLP promotor drives GFP expression (PLP-GFP mice^32^), about 90% (90.09% ± 2.6%; *n* = 13, *N* = 4) of the CC1 positive cells also expressed PLP-GFP (p15-p16, Figures 2A1–2A5) and all PLP-GFP expressing oligodendrocytes were CC1 positive. Only a few CC1 positive cells lacked co-localization with PLP-GFP (9.91% ± 2.6%) and all PLP-GFP positive oligodendrocytes were CC1 positive (Figure 2A5). To decide whether CC1 positive cells lacking PLP-GFP are NG2 glia, thalamic slices from NG2-YFP mice were stained against CC1 and YFP. Indeed, 25% (25.32% ± 5.28%; *n* = 7, *N* = 3) of the CC1 positive cells were also NG2-YFP positive (Figures 2B1–2B5) and 34% (34.60% ± 1.12%) of NG2-YFP expressing cells were stained by CC1 antibody. Two-thirds (65.40% ± 1.12%) of the NG2-YFP expressing cells lacked co-localization with CC1, and 74.68% ± 5.28% of the CC1 positive cells were devoid of NG2-YFP (Figure 2B5). Given that thalamic NG2 glial cells are not gap junction coupled,^7^ this data confirmed CC1 as useful marker for oligodendrocytes among the biocytin-positive cells in the barreloids.

### Characterization of the cellular composition of panglial coupling networks

**Figure 3:**
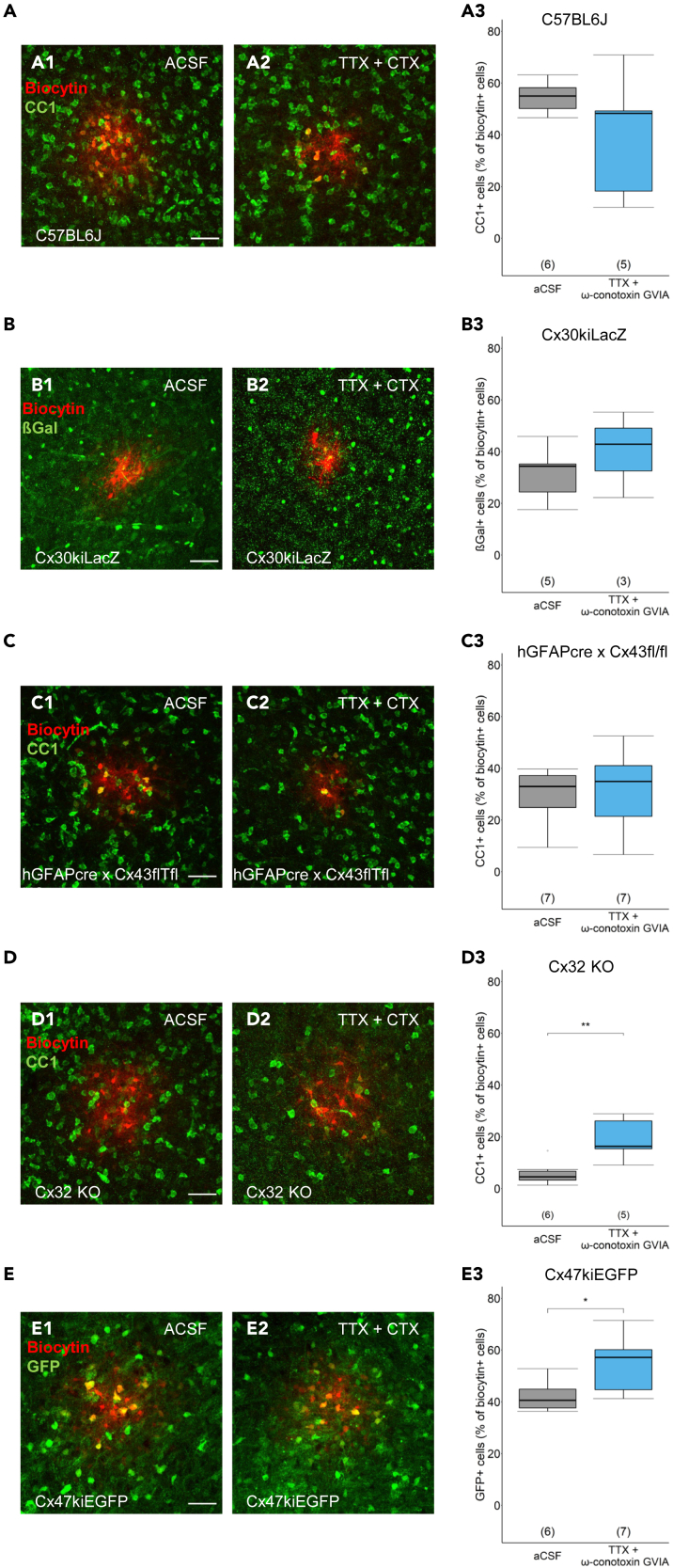
Characterization of panglial coupling networks in barreloid fields (A–E) (A1 and A2–E1 and E2) Representative MIPs of biocytin-filled astrocytes and histochemical staining against CC1, βGal, or GFP as indicated. Scale bar: 50 μm. (A3–E3) Quantification of the proportion CC1, βGal, or GFP positive cells among the biocytin labeled cells in aCSF and aCSF supplemented with TTX (0.5 μM) + CTX (0.5 μM). Data are displayed as boxplots representing median and quartiles. Number of mice is given in brackets. Two sample t test. Stars indicate statistical significance. ∗*p* < 0.05, ∗∗*p* < 0.01.

Thalamic astrocytes and oligodendrocytes equally contribute to panglial gap junction networks^7^ and are crucial for proper glial-neuron crosstalk.^26^^,^^33^ We asked whether after genetic ablation of glial connexins, neuronal activity affects the cellular composition of these networks. In wild-type mice, the biocytin labeled coupling networks contained 55% CC1 positive oligodendrocytes, and this proportion did not change after TTX + CTX treatment (Table 3; Figure 3A). In Cx30kiLacZ mice, expression of the reporter βGal was used to identify astrocytes in the biocytin network. In the control group, 34.4% were astrocytes and in this proportion remained unchanged after TTX + CTX incubation (Table 3; Figure 3B). As mentioned previously, some astrocytes lacking Cx30 were completely uncoupled after TTX + CTX application (Figure 1D2); these cells were not considered in the network analysis shown in Figure 3B3. Similarly, no differences in network composition were found in the barreloids of hGFAPcre x Cx43 fl/fl mice after deprivation of neuronal activity. In control solution: 33% of the biocytin-positive cells were CC1 positive and this proportion did not change after TTX + CTX application (Table 3; Figure 3C). Since Cx32 is the dominating oligodendroglial connexin isoform in the thalamus,^7^ CC1 positive cells were rarely found in thalamic coupling networks of Cx32 KO mice, but its proportion increased after adding TTX + CTX (Table 3; Figure 3D). A similar increase in the proportion of oligodendrocytes in the coupled networks after deprivation of neuronal activity was observed in barreloids of Cx47kiEGFP mice. In control solution, 40.6% of the biocytin positive cells also expressed EGFP, compared to 57.1% after application of TTX + CTX (Table 3; Figure 3E). In conclusion, in the barreloids of mice lacking oligodendroglial connexins, the composition of the coupling networks was dependent on neuronal activity, whereas this effect was not observed in mice deficient for astroglial connexin isoforms.

**Table 3:** Proportion (%) of CC1- or GFP- positive oligodendrocytes and LacZ-positive astrocytes (in case of Cx30kiLacZ mice) in panglial gap junction networks in thalamic barreloids under control conditions and after application of 0.5 μM TTX +0.5 μM CTX

Mouse line	ACSF	ACSF +TTX + CTX	*p* value
C57BL6	55.0 (50.2–58.2) *n* = 11, *N* = 6	48.3 (18.3–49.2) *n* = 8, *N* = 5	0.18 two-sample t test
Cx30kiLacZ	34.4 (24.4–35.2) *n* = 6, *N* = 5	42.9 (32.5–49.1) *n* = 3, *N* = 3	0.40 two-sample t test
hGFAPcre x Cx43 fl/fl	33.0 (24.8–37.2) *n* = 11, *N* = 7	34.9 (21.4–41.1) *n* = 13, *N* = 7	0.81 two-sample t test
Cx32 KO	4.6 (3.3–6.8) *n* = 9, *N* = 6	16.3 (15.4–26.2) *n* = 6, *N* = 5	0.008 two-sample t test
Cx47kiEGFP	40.6 (37.7–44.9) *n* = 10, *N* = 6	57.1 (44.7–60.2) *n* = 8, *N* = 7	0.04 two-sample t test

Data are given as median and interquartile range (quartile 25% - quartile 75%), *n* = cell numbers, *N* = number of animals.

### Connexin transcript expression in knockout mice

The aforementioned data demonstrate the impact of neuronal activity on panglial coupling, but connexin expression and coupling might also be affected by interactions between isoforms.^33^^,^^34^ To test for potential compensatory regulation, we compared the thalamus of C57BL6/J mice (control) with that of mice lacking Cx30, Cx32, Cx43, or Cx47 for expression of the respective remaining connexin isoforms on the transcript level. While expression of Cx43 or Cx47 was not altered in Cx KO mice, Cx30 was upregulated in mice deficient for oligodendroglial Cx32 or Cx47, and downregulated if Cx43 was lacking (Figure S2A). Upregulation of Cx32 was found in Cx47kiEGFP mice (Figure S2B). Thus, the unexpected finding of an increased proportion of oligodendrocytes in the coupling network after deprivation of neuronal activity in mice lacking Cx32 or Cx47 (Figures 3D and 3E) might have been due to compensatory upregulation of Cx30 and enhanced Cx30:Cx47 or Cx30:Cx32 panglial coupling.

### Reduced sensory input impairs panglial coupling in the barreloids *in vivo*

**Figure 4:**
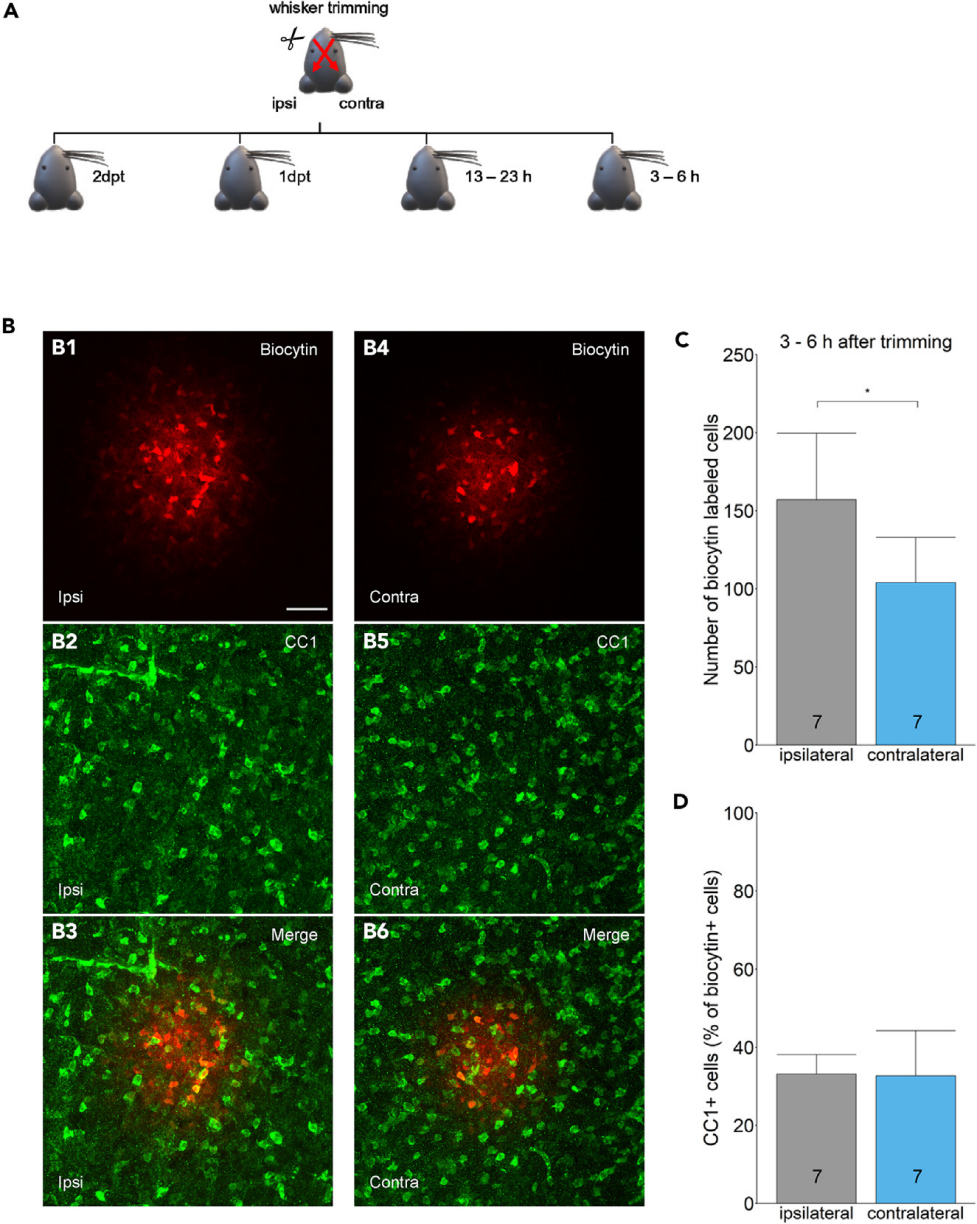
Sensory deprivation impairs panglial coupling *in vivo* (A) Schematic of unilateral whisker trimming. The trimmed side of the snout is represented in the contralateral thalamus (top, red arrows). Coupling analyses were performed after trimming at time points as indicated (dpt, days post trimming). (B) Individual astrocytes within barreloids, ipsi-, and contralaterally to the trimmed side were filled with biocytin for 20 min. Representative MIPs of biocytin-positive (B1 and B4) and CC1-positive cells (B2 and B5) are shown, together with the merged pictures (B3 and B6), 3–6 h after trimming (C57BL6/J mice). Scale bar: 50 μm. (C) Quantitative analyses in C57BL6/J mice (p13-15) 3–6 h after trimming revealed a lower number of biocytin-positive cells, i.e., reduced coupling efficiency, in the contralateral thalamus. (D) The proportion of CC1-positive oligodendrocytes among the biocytin labeled cells was not affected 3–6 h after trimming. Bar graphs represent mean ± SD. Number of mice is given in bar graph. Two sample t test or Welch two sample t test. ∗*p* < 0.05.

The finding that coupling efficiency *in situ* depends on neuronal activity prompted us to test whether deprivation of activity also affects glial coupling *in vivo*. In juvenile mice, whisker trimming reduces the sensory input to the somatosensory cortex via the barreloids.^20^ Coupling analyses were performed between p13 to p17, a period in which barreloids are visible in acute tissue slices.^17^ Mice retained whiskers on one side of the snout (ipsilateral, control) whereas the other side, which is represented in the contralateral thalamus, was trimmed (Figure 4A). First, mice were sacrificed one (1dpt) or two-days-post-trimming (2dpt). Acute brain slices were prepared and the coupling efficiency in the barreloids was examined. Under these conditions, trimming did not affect coupling (1dpt: ipsilateral 183.1 ± 43.6 biocytin-positive cells, *n* = 13, *N* = 10; contralateral 173.0 ± 54.7 cells, *n* = 12, *N* = 10, *p* = 0.65; 2dpt: ipsi 172.8 ± 45.8 cells, *n* = 9, *N* = 7; contra, 159.6 ± 28.3 cells, *n* = 12, *N* = 7; *p* = 0.53, two sample t-test) (Figure S3). Next, we looked at an earlier time point and counted biocytin-positive cells 13–23 h after trimming, which again did not reveal differences in coupling between ipsi- (161.4 ± 46.7 cells, *n* = 11, *N* = 4) and contralateral sides (118.1 ± 15.9, *n* = 9, *N* = 4; *p* = 0.13, two sample t test) (Figure S3). Finally, we tried to mimic the experimental setting *in situ* where coupling was affected 3–4 h after TTX + CTX application. Indeed, performing coupling analysis in the barreloids at 3–6 h after whisker trimming revealed a significant decrease in coupling efficiency (contralateral 103.9 ± 28.9, *n* = 12, *N* = 7 vs. ipsilateral (157.0 ± 42.6, *n* = 12, *N* = 7; *p* = 0.018, two sample t test) (Figures 4B and 4C). Sham manipulation (whisker clasping) did not affect coupling efficiency 3–6 h later (ipsilateral 114.8 ± 25.6, *n* = 10, *N* = 6; contralateral 106.0 ± 40.0, *n* = 9, *N* = 6; *p* = 0.66, two sample t test) (not shown). Interaction effect of trimmed side of snout (left vs. right) on the main effect of biocytin network size was ruled out (checked with ANOVA), and daytime did also have no effect.

We also addressed the question whether sensory deprivation affects the cell composition of the panglial network *in vivo*. Three to 6 h after trimming, no changes were observed in the proportion of CC1 positive cells within the biocytin labeled network, neither in trimmed mice (ipsi: 33.1 ± 5.0%, *n* = 11, *N* = 7; contra: 32.7 ± 11.6%, *n* = 12, *N* = 7; *p* = 0.92, Welch two sample t test) (Figures 4B and D) nor in sham control mice (ipsi: 29.4 ± 7.3%, *n* = 10, *N* = 6; contra: 25.7 ± 10.7%, *n* = 9; *N* = 6; *p* = 0.49, two sample t test).

In conclusion, short-term sensory deprivation through whisker trimming recapitulated the effect of neuronal deprivation *in situ* on the efficiency of panglial coupling, while the proportion of oligodendrocytes in the panglial coupling network remained unaffected.

## Discussion

Previous work revealed that glial gap junction coupling critically contributes to proper brain function. For example, loss of astrocyte coupling due to deletion of Cx30 and/or Cx43 impaired the uptake and redistribution of K^+^ and glutamate, generated aberrant neuronal activity, and impaired spatial learning and memory.^35^^,^^36^^,^^37^^,^^38^ Oligodendrocytes interact closely with axons and support their function by supplying energy-rich substrates through myelin ensheathment.^39^^,^^40^^,^^41^ However, both astrocytes and oligodendrocytes also supply neurons with glucose, pyruvate and lactate via their coupling networks, to ensure long-term functionality of axons and synapses.^26^^,^^27^^,^^28^ The latter studies have shown that impairment of junctional communication profoundly influences synaptic activity and myelination. Thalamic oligodendrocytes and astrocytes express glutamine synthetase.^7^ This enzyme might also be distributed through the panglial network to support synaptic transmission^42^ and behavior, as in spinal cord where oligodendroglial GS is important for glutamine supply to motor neurons and control of forelimb force.^43^ N-methyl-D-aspartate (NMDA) receptor signaling in oligodendrocytes and glutamate transporter activation in astrocytes may be involved in regulating these forms of cellular communication.^44^^,^^45^

Whisker-specific patterning of thalamocortical terminals in rodents emerges during the first days after birth. Each barreloid and barrel receives sensory information from a single whisker. The principal sensory nucleus of the trigeminal nerve is targeted by axons from the trigeminal ganglion and after crossing of axons to the contralateral hemisphere, information is transferred into the thalamic VPM where in mice barreloids become visible at p3. Two days later, patterning and topographic organization into barrels appears in the primary somatosensory cortex,^46^ which is dependent on intrinsic molecular cues and involves signaling to NMDA receptors^47^ and mGluR5.^48^^,^^49^ We show here that unilateral whisker trimming recapitulates *in vivo* effects of TTX + CTX application on coupling in the slice preparation. While the players modulating gap junctions in the barreloids are yet unknown, glial networks in neocortical barrels were thought to be influenced by extracellular matrix molecules and proteoglycans.^50^ No plastic changes upon sensory deprivation were found in the VPM in earlier studies, but they were often performed in adult animals and focused on long term plasticity.^25^^,^^51^ After clipping of whiskers, thalamocortical axons from the VPM are shortened, independent of animals’ age.^22^^,^^23^ Our data reveal another type of plasticity within the VPM, induced by short-term deprivation in young mice. The changes in glial coupling efficiency occurred within an early, short time window after trimming and reversed over time. The question of how these glial changes influence the transfer of information from the thalamus to the sensorimotor cortex should be the subject of future studies.

While glial coupling is crucial for maintenance of neuronal activity, in the present study we asked whether, conversely, as a kind of possible feedback mechanism, transfer of metabolic substrates through coupling might also be influenced by neuronal activity. Indeed, such a relation exists in olfactory glomeruli where inhibition of neuronal activity by TTX or sensory deprivation entailed reduced astrocytic coupling through modulation of Cx30-formed channels.^29^ Coupling in the barreloids is reduced after application of TTX and CTX.^17^ Because of its dominant expression in thalamic astrocytes^7^ we wondered whether in this brain region, Cx30 channel function is also activity dependent. After Cx30 deletion, thalamic astrocytes displayed reduced tracer spread, but this remaining coupling was still sensitive to neuronal firing. In contrast, in barreloids of mice lacking Cx32 or Cx47, the panglial networks were no longer affected by neuronal activity, suggesting that junctional channels in oligodendrocytes are the target. In the thalamus of Cx32^−/−^;Cx47^EGFP(+/−)^ mice the proportion of oligodendrocytes in the panglial coupling network is strongly reduced,^26^ and it is even lower in Cx32 knockout mice (this study). Obviously, heterotypic coupling of Cx47 with Cx43, i.e., between oligodendrocytes and astrocytes, is unable to restore coupling in Cx32 KO mice. Together, our data confirm the view that in the thalamus, Cx32 is the dominant oligodendroglial isoform and astrocyte-to-oligodendrocyte coupling is predominantly mediated through Cx30:Cx32 channels.^7^

The question of through which mechanism(s) neuronal activity influences glial coupling remains open. Neurotransmitters, cytokines, or other endogenous molecules might be candidates. In the cerebellum, the coupling efficiency of Bergmann glia is decreased by activation of 2-Amino-3-(3-hydroxy-5-methyl-isoxazol-4-yl)propanoic acid (AMPA) receptors, probably through receptor-mediated Ca^2+^ influx.^52^ A similar scenario is conceivable in the VPM where the sensory fibers to thalamocortical neurons are glutamatergic. Thalamic astrocytes also express functional AMPA receptors,^53^ and Ca^2+^ elevations have been observed in these cells after mGluR activation.^54^ Whether oligodendrocytes in the VPM are sensitive to glutamate is not known. In the optic nerve, oligodendrocytes express Ca^2+^ permeable NMDARs on their myelin sheaths, which besides modulation of GLUT1 trafficking and axonal energy supply^41^^,^^55^ might also affect coupling.

In control mice and mice lacking astroglial connexins, coupling efficiency was reduced when dampening neuronal firing, but the proportion of astrocytes vs. oligodendrocytes within the network remained unchanged. This was different in Cx32 KO and Cx47kiEGFP mice where the proportion of oligodendrocytes within the coupling became larger when TTX and CTX were added. Since overall coupling efficiency did not change in these mice, as judged by tracer injection, it is likely that oligodendrocytes were preferred to couple in the ko mice, while astrocyte coupling was impaired. This could mean that under conditions of neuronal suppression, as a kind of homeostatic scaling, the respective remaining isoform is compensatorily upregulated to keep oligodendrocytes in the panglial network.

Taken together, we show that in the ventrobasal thalamus, the efficiency of panglial coupling is regulated by synaptic activity. Systematic analyses of mice lacking individual connexin isoforms revealed that the target of this functional modulation is not astrocytes but the oligodendroglial connexins Cx32 and Cx47. Under conditions of suppressed neuronal activity, the proportion of oligodendrocytes within the coupling network increased if Cx32 or Cx47 were deleted. In Cx30 ko mice, the size of the coupling networks was reduced, confirming the assumption that this isoform dominates in thalamic astrocytes. Finally, we show that during a critical time window, sensory deprivation reduces thalamic coupling also *in vivo*. Together with our previous results^26^ the current findings indicate that neuronal activity and provision of energy metabolites through panglial coupling are interdependent events regulated in a bidirectional manner.

### Limitations of the study

In this work, we have shown that the efficiency of panglial coupling is regulated by synaptic activity, and that oligodendroglial Cx32 and Cx47 are the targets of this modulation. Importantly, we show that during a critical time window, sensory deprivation through whisker trimming reduces the efficiency of the glial transfer networks also *in vivo*. While our data reveal a previously unknown type of plasticity within the VPM, the important question of how these glial changes influence the transfer of information from the thalamus to the sensorimotor cortex remains to be addressed in future studies.

## Resource availability

### Lead contact

Further information and requests for resources and reagents should be directed to and will be fulfilled by the Lead Contact, Christian Steinhäuser (christian.steinhaeuser@uni-bonn.de).

### Materials availability statement

This study did not generate new unique reagents.

### Data and code availability

• All data in this paper will be shared by the lead contact upon request.
• This paper does not report original code.
• Any additional information required to reanalyze the data reported in this paper is available from the lead contact upon request.

## Acknowledgments

We thank Thomas Erdmann and Dario Tascio for excellent technical assistance. This work was supported by 10.13039/501100001659Deutsche Forschungsgemeinschaft (STE 552/4, KE329/28) and a stipend from the Else-Kröner-Fresenius foundation (EKFS Q-614.1154, to P.B.).

## Author contributions

Conceptualization, C.S. and H.K.; methodology, P.B., R.J., R.T.N., M.O., and G.S.; investigation, P.B., A.B., L.Z., L.L., and G.S.; software, R.J.; supervision, G.S. and C.S.; writing – original draft, P.B., G.S., and C.S.; writing – review and editing, PB, G.S., H.K., and C.S.; funding acquisition, C.S. and H.K.

## Declaration of interests

The authors declare no competing interests.

## STAR★Methods

### Key resources table

**Table:** 

REAGENT or RESOURCE	SOURCE	IDENTIFIER
**Antibodies and biotin-binding proteins (streptavidin)**		

Chicken anti-GFP	Abcam	Cat# ab13970; RRID:AB_300798
Rabbit anti-β-Gal	Invitrogen	Cat# A-11132; RRID:AB_221539
Mouse anti-APC mAB	Calbiochem	Cat# OP80
Goat anti-chicken Alexa Fluor 488	Invitrogen	Cat# A-11039; RRID:AB_142924
Goat anti-mouse Alexa Fluor 488	Invitrogen	Cat# A-11029; RRID:AB_2534069
Goat anti-mouse Alexa Fluor 647	Invitrogen	Cat# A-21235; RRID:AB_2535805
Goat anti-rabbit Alexa Fluor 488	Invitrogen	Cat# A-11034; RRID:AB_2576217
Goat anti-rabbit Alexa Fluor 647	Invitrogen	Cat# A-21244; RRID:AB_2535812
Streptavidin, Alexa Fluor 647 conjugate	Invitrogen	Cat# S-32357; RRID:AB_2336066
Streptavidin, Alexa Fluor 488 conjugate	Invitrogen	Cat# S-11223; RRID:AB_2315383
Streptavidin, Cy3 conjugate	Sigma-Aldrich	Cat# S6402

**Chemicals, peptides, and recombinant proteins**		

Tetrodoxin citrate	Abcam	Cat# ab120055; CAS: 18660-81-6
ω-conotoxin GVIA	Sigma-Aldrich	Cat# C9915; CAS: 106375-28-4
ω-conotoxin GVIA	Alomone Labs	Cat#C-300; CAS: 106375-28-4
Biocytin	Sigma-Aldrich	Cat# B4261; CAS: 576-19-2
Texas Red Dextran MW 3000	Invitrogen	Cat# D-3328
Sulforhodamine 101	Sigma-Aldrich	Cat# S7635; CAS: 60311-02-6
Hoechst 33342	Invitrogen	Cat# H1399; CAS: 23491-52-3
Sulforhodamine 101	Sigma-Aldrich	Cat# S7635; CAS: 60311-02-6

**Experimental models: Organisms/strains**		

Mouse: C57BL6/J (Charles River, Sulzfeld; Germany)	Colony maintained at the facility of the University of Bonn	N/A
Mouse: PLP-GFP (Fuss et al.^32^)	Colony maintained at the facility of the University of Bonn	N/A
Mouse: NG2kiYFP (Karram et al.^56^)	Colony maintained at the facility of the University of Bonn	N/A
Mouse: Cx30kiLacZ (Teubner et al.^57^)	Colony maintained at the facility of the University of Bonn	N/A
Mouse: Cx32 KO (Nelles et al.^58^)	Colony maintained at the facility of the University of Bonn	N/A
Mouse: hGFAPcre x Cx43 fl/fl (Tress et al.^33^)	Colony maintained at the facility of the University of Bonn	N/A
Mouse: Cx47kiEGFP (Tress et al.^33^)	Colony maintained at the facility of the University of Bonn	N/A

**Software and algorithms**		

ImageJ	NIH	https://fiji.sc/or https://imagej.nih.gov/ij/; RRID:SCR_002285
Igor Pro 7.0	WaveMetrics	https://www.wavemetrics.com RRID:SCR_000325
R v3.5.2	R Core Team (2018)	http://www.R-project.org/RRID:SCR_001905
LAS AF/LAS X	Leica	https://www.leica-microsystems.com/products/microscope-software/p/leica-las-x-ls/RRID:SCR_013673
MC Stimulus II	Multi Channels Systems	https://www.multichannelsystems.com/software/mc-stimulus-ii
Tida v5.24	HEKA	https://www.heka.com/RRID:SCR_014582

### Experimental model

#### Mice

Experiments were performed in wildtype C57BL6/J (Charles River, Sulzfeld, Germany or bred in-house), Cx30kiLacZ,^57^ Cx32KO,^58^ Cx47kiEGFP,^33^ hGFAPcre x Cx43fl/fl,^33^ myelin proteolipid protein-GFP (PLP-GFP)^32^ and NG2kiYFP^56^ mice of either sex. Age of mice was between postnatal days (p) p14-17, if not indicated otherwise; for whisker trimming experiments mice of p12-17 were used. Mice were kept under standard housing conditions with 12 h day/night shift and food/water *ad libitum*. All experiments were carried out in accordance with local, state, and European regulations (animal license 81-02.04.2020.A226).

### Method details

#### Slice preparation

Mice were anesthetized with isoflurane (Primal Healthcare, Morpeth, UK) and decapitated. Brains were dissected, cut into two hemispheres, and the cutting surface was placed on a 30° block. A small piece of the ventral part was cut off vertically at the angular edge, and the hemisphere was glued with the ventral cutting surface facing down on a 5° block, rostral part pointing downwards. This preparation resulted in a cutting plane tilted up 5° anteriorly and 30° laterally from the horizontal allowing visualization of barreloids in acute brain slices.^17^ The specimen holder was transferred to a chamber filled with ice-cold, preparation solution (87 mM NaCl, 2.5 mM KCl, 1.25 mM NaH_2_PO_4_, 25 mM NaHCO_3_, 7 mM MgCl_2_, 0.5 CaCl_2_, 25 mM glucose, 61 mM sucrose) bubbled with carbogen (95% O_2_/5% CO_2_) and the tissue was cut into 200 μm thick horizontal slices using a vibratome (VT1200S, Leica Microsystems). Subsequently, slices were transferred into preparation solution for 20 min (35°C, gassed with carbogen) and then incubated in artificial cerebrospinal fluid (aCSF: 1.25 mM NaH_2_PO_4_, 126 mM NaCl, 3 mM KCl, 2 mM MgSO_4_, 2 mM CaCl_2_, 10 mM glucose, 26 mM NaHCO_3,_ gassed with carbogen) (pH 7.4), supplemented with 1 μM sulforhodamine 101 (SR101; S7635, Sigma-Aldrich, Munich, Germany) at 35°C for 20 min. Afterward, the slices were stored in aCSF gassed with carbogen at room temperature. For activity-dependent coupling experiments slices were incubated either in aCSF with 0.5 μM tetrodotoxin (TTX) (Abcam, ab120055) and 0.5 μM CTX (Sigma Aldrich, C9915; C-300, Alomone Labs, Jerusalem, Israel) or just with aCSF (room temperature, 3–4 h). Finally, slices were checked for visible barreloids on an upright microscope (Eclipse E600FN, Nikon, Germany). If not stated otherwise, chemicals were purchased from AppliChem GmbH (Darmstadt, Germany), Fluka and Carl Roth (Karlsruhe, Germany).

#### Electrophysiological recordings

Slices were transferred to a recording chamber and continuously perfused with carbogen-gassed aCSF with or without 0.5 μM TTX and 0.5 μM CTX (room temperature). Cells were visualized using an upright microscope equipped with infrared differential interference contrast using 60× water immersion objectives (Eclipse E600FN, Nikon). Patch pipettes were fabricated from borosilicate glass capillaries (Science Products, Hofheim, Germany) by a horizontal puller (DMZ Zeitz-Puller, Zeitz, Martinsried, Germany) and had a resistance of 3–6 MΩ. Pipette solution was composed of (in mM): 130 K-gluconate, 1 MgCl_2_, 3 Na_2_ -ATP, 10 2-[4-(2-hydroxyethyl)piperazin-1-yl]ethanesulfonic acid (HEPES), 10 ethyleneglycol-bis(2-aminoethylether)-N,N,N,N′-tetra acetic acid (EGTA), supplemented with 0.5% N-biotinyl-l-lysine (Biocytin, Sigma Aldrich, B4261) and 0.1% Texas Red Dextran (3000 MW, Invitrogen, D3328) (pH 7.2). In this study, recordings were exclusively obtained from astrocytes located in the center of the barreloid fields. They were identified by their SR101 fluorescence, their morphological characteristics, passive current patterns, very negative resting membrane potentials and a low membrane resistance. For the analysis of gap junction coupling, an astrocyte within the barreloid field was filled with biocytin and Texas Red Dextran by the patch pipette (20 min, room temperature). Recordings were performed in the whole cell configuration with a holding potential of −80 mV; liquid junction potential (−7 mV) was corrected online. Currents were recorded by applying 10 mV voltage steps every 10 min to determine input and series resistance. The following exclusion criteria have been defined: resting potential > −60 mV, membrane resistance >10 MΩ, series resistance >20 MΩ. An EPC9 amplifier (Heka, Lambrecht, Germany) was used; signals were filtered at 3 or 10 kHz and sampled at 10 or 30 kHz. TIDA 5.25 software (HEKA) and Igor Pro 6.37 software (WaveMetrics, Lake Oswego, OR, USA) were used for data acquisition and analysis. After recording, slices were fixed in 4% paraformaldehyde (PFA) in phosphate buffered saline (PBS) (pH 7.4, 4°C, overnight) and stored in 0.1 M PBS.

#### Visualization of tracer coupling

To assess cell-to-cell coupling after recording, biocytin spread within the glial networks was visualized. Therefore, slices were washed (3x with 0.1 M PBS, 10 min each) and subsequently incubated with 2 or 5% Triton X-100 (Sigma-Aldrich, Munich, Germany) and 10% normal goat serum (NGS) (Merck Millipore, Darmstadt, Germany) in PBS (2 h, room temperature) to block unspecific bindings sites. Afterward, slices were incubated with primary antibodies in PBS containing 2% NGS and 0.1% Triton over night at 4°C. The following antibodies were used: 1:600 Alexa Fluor 647 streptavidin conjugate (Invitrogen, S32357); 1:600 Alexa Fluor 488 streptavidin conjugate (Invitrogen, S11223); 1:300 streptavidin conjugated Cy3 (Sigma S6402); 1:500 chicken-*anti*-GFP (Abcam ab13970); 1:500 rabbit-*anti*-β-Gal (Invitrogen, A-11132); 1:500 mouse-*anti*-APC (CC-1) mAb (Calbiochem, OP80, monoclonal). The next day, slices were washed (3x times in PBS, 10 min each), and then incubated in PBS with 2% NGS and 0.1% Triton together with the corresponding secondary antibody (room temperature, 2 h). The following secondary antibodies were used: 1:500 goat-*anti*-chicken Alexa Fluor 488 (Invitrogen A-11039); 1:500 goat-*anti*-rabbit Alexa Fluor 488 (Invitrogen A-11034); 1:500 goat-*anti*-mouse Alexa Fluor 488 (Invitrogen A-11029); 1:500 goat-*anti*-mouse Alexa Fluor 647 (Invitrogen A-21235); 1:500 goat-*anti*-rabbit Alexa Fluor 647 (Invitrogen A-21244). Since SR101 is not fixable it did not interfere with biocytin visualization. The slices were washed with PBS (3x, 10 min each), stained with Hoechst 33342 (1:100 in dH_2_O) (Invitrogen, H1399) (10 min, room temperature) and washed again with PBS (3x, 10 min each). Then, slices were mounted on microscope slides covered with cover glasses using Aqua-Poly/Mount (Polysciences Europe, 18606) and stored at 4°C.

#### Immunohistochemistry

Mice were anesthetized and transcardially perfused with ice-cold PBS followed by 4% ice-cold PFA in PBS.^17^ Dissected brains were postfixed in 4% PFA overnight and transferred to PBS (4°C). Brains were cut on a vibratome (VT1200 S, Leica Microsystems) into 40 μm thick horizontal slices with the cutting plane tilted up 5° anteriorly and 30° laterally from the horizontal and stored until staining in PBS supplemented with NaN_3_ (100 mg/L). They were washed (PBS, 3x, 10 min each) and blocked with 10% NGS (Merck Millipore, Darmstadt, Germany) and 0.5% Triton X-100 (Sigma-Aldrich, Munich, Germany) in PBS (2 h, room temperature). After washing (PBS, 3x, 10 min each), slices were incubated with primary antibodies in PBS containing 5% NGS and 0.1% Triton X-100 (4°C, overnight). The following antibodies were used: 1:500 chicken-*anti*-GFP (Abcam ab13970); 1:500 mouse-*anti*-APC (CC-1) mAb (Calbiochem, OP80, monoclonal). The next day, slices were washed (PBS, 3x, 10 min each) and incubated in PBS with 5% NGS and 0.1% Triton containing the corresponding secondary antibodies (room temperature, 2 h): 1:500 goat-*anti*-chicken Alexa Fluor 488 (Invitrogen A-11039); 1:500 goat-*anti*-mouse Alexa Fluor 647 (Invitrogen A-21235). The slices were washed (PBS, 3x, 10 min each) and stained with 1:100 Hoechst 33342 (Invitrogen H1399) in dH_2_O (10 min, room temperature). After wash (PBS, 3x, 10 min each), slices were mounted on microscope slides covered with cover glasses using Aqua-Poly/Mount (Polysciences Europe 18606) and stored at 4°C.

#### Whisker trimming

Trimming experiments were performed with C57Bl6/J mice between p12 and p16, a period in which the barreloids are visible in the VPM, whisking is active and eyes are open.^20^ Before trimming, the animals were allowed to get familiar with the experimenter’s hand, microscope stage and trimming devices. Animals were carefully fixed by hand during trimming, and macrovibrissae of the rows A-E were trimmed on one side of the snout down to follicle base (length <1 mm) under microscopic control (Stemi 2000 with eyepiece PL 10x/23 spectacle focusing, Zeiss) using microsurgical scissors. The trimmed snout side was alternated among litter. Sham control animals were handled similarly, except their whiskers were not trimmed but mimicked to be trimmed using flat scissors or a pair of tweezers. After trimming, animals were kept together with their mother and littermates in an enriched cage (cardboard tube, square) to enhance whisker-based exploration.^22^ Mice were not re-trimmed, and gap junction coupling was investigated at different time points after trimming (3–6 h, 13–23 h, 1dpt and 2dpt). Time after trimming refers to the time point when a cell was electrophysiologically characterized and filled with the tracer. Mice with damaged or not normally developed whisker architecture, as a consequence of natural whisker trimming by littermates or the parents,^59^^,^^60^ were excluded. Whisker pad architecture is always represented in the contralateral thalamic barreloids. Thus, ipsilateral slices of the VPM represent the untrimmed control side, and contralateral slices the trimmed side of the snout. Data were always compered in corresponding ipsi-vs. contralateral slices.

#### Preparation of brain tissue for RT-qPCR

To examine connexin expression, reverse transcription-semiquantitative polymerase chain reaction (RT-qPCR) was performed. Tissue samples of the VPM of the thalamus from mice of both genders (p5 – 45) were dissected from 200 μm thick brain slices. Total RNA was isolated with Trizol (Ambion), precipitated with isopropanol, washed and dissolved in 10 μL DEPC-treated water. Genomic DNA was removed by DNase treatment in a mixture containing PCR buffer, 2.5 mm MgCl_2_, 10 mm DTT (all Invitrogen), 20 U DNaseI (Roche), and 40 U RNase inhibitor (Promega; final volume 20 μL; incubation at 37°C for 30 min). Subsequently, mRNA was isolated using oligo(dT)25-linked Dynabeads (Invitrogen). Dynabeads suspended in lysis buffer (20 μL) were added to the reaction tube. After wash (100 μL), beads with the adherent mRNA were suspended in DEPC-treated water (20 μL), frozen, and stored at −20°C.

#### RT-qPCR

The reaction mix of the RT reaction contained first strand buffer, dithiothreitol (10 mM), deoxynucleoside triphosphates (4 × 250 μM; all from Thermo Fisher Scientific), random hexamer primers (50 μM; Roche Diagnostics), RNase inhibitor (40 U; Promega), and SuperScript III Reverse Transcriptase (200 U; Thermo Fisher Scientific). The reaction mix (21 μL) and mRNA (20 μL) were incubated at 37°C for 1 h. The reaction volume for real-time PCR contained PCR mastermix (Takyon, Eurogentec), TaqMan gene expression assay, and 1 μL cDNA; final volume was 12.5 μL. The reaction mix without cDNA served as a negative control. Samples were incubated at 50°C (2 min) and denaturated at 95°C (10 min), followed by 50 cycles of PCR (denaturation at 95°C for 15 s; primer annealing and extension at 60°C for 1 min). Fluorescence intensity was readout during each annealing/extension step (CFX384 PCR System, Bio-Rad Laboratories). Information about the TaqMan gene expression assay is listed in Table 1. Relative gene expression was determined by comparing threshold cycle values of the target genes with those of the reference gene β-actin at the same fluorescence emission intensity. The relative quantification of different genes was determined according to the equation:

(Equation 1) $$X_{\text{Cx}}\;/X_{\beta -\text{actin}}=E_{\beta -\text{actin}}^{\text{CT}\beta -\text{actin}}/\;E_{\text{Cx}}^{\text{CTCx}}$$

where X_Cx_/X_β-actin_ is the gene ratio, and CT_β-actin_ and CT_Cx_ are the threshold cycle numbers of β-actin and Cx, and E_β-actin_ and E_Cx_ the amplification efficiencies of β-actin and Cx genes, respectively.

### Quantification and statistical analysis

Images were acquired with a confocal laser scanning microscope (TCS SP8 Leica) using 10x and 20× objectives. Individual optical planes were taken at an interval of 2 μm (gap junction coupling) or 1 μm (co-localization with cell markers). Biocytin-positive cells were counted through the z stack with the cell counter plugin of Fiji/ImageJ. Data were averaged per mouse and finally averaged over the number of mice. To identify oligodendrocytes or astrocytes in the biocytin-labeled network, tissue was co-stained with respective cell type-specific markers. In Cx30kiLacZ mice, uncoupled biocytin-positive cells were not considered in the analysis. To test whether anti-CC1 antibody is suited to identify oligodendrocytes in the thalamus, co-localization with fluorescent cells in z-planes of PLP-GFP and NG2-YFP mice was quantified. Counting was performed by two different persons blinded to the experimental condition. Images displayed in the figures are maximum intensity projections (MIPs) of a representative region of interest (ROI). Hoechst staining and Texas Red Dextran are not shown in the figures. Data were analyzed using R software (R Core Team (2020), R Foundation for Statistical Computing, Vienna, Austria, version 4.0.2). Homogeneity of variance between groups was tested using Levene’s test. Normal distribution was checked with Shapiro-Wilk test. In case of violation of normality, data were transformed using Tukey’s Ladder of Powers^61^ or an appropriate non-parametric test was used. A two sample t-test with or without Welch correction for equal or diverse variance and Wilcoxon rank-sum test was applied for comparison of two independent groups. Interaction effects of variables were checked by running two-way analysis of variance (ANOVA). Data are given as mean ± standard deviation (SD) or as boxplots representing median (central line), first and third quartiles (25^th^ and 75^th^ percentile). Whiskers extend to the highest and lowest observation within 1.5 times the interquartile range (IQR). Extreme outliers (first quartile minus 3 times IQR or third quartile plus IQR) were excluded from the analysis. Number of mice (N) per condition is given in the figures, number of slices or cells (n) is given in figure legends or results. Differences in means were regarded as significant at *p* < 0.05.
