# Supplementary material for: Activity dependent modulation of glial gap junction coupling in the thalamus
Source: iScience. 2024 Sep 26;27(10):111043. doi: 10.1016/j.isci.2024.111043 (PMC11491719; doi:10.1016/j.isci.2024.111043)
Supplement: Document S1. Figures S1–S3 [file mmc1.pdf]

iScience, Volume *27*

Supplemental information

Activity dependent modulation of glial gap junction coupling in the thalamus

Paula Baum, Anna Beinhauer, Lara Zirwes, Linda Loenneker, Ronald Jabs, Rajeevan T. Narayanan, Marcel Oberlaender, Gerald Seifert, Helmut Kettenmann, and Christian Steinhäuser

Supplemental Information

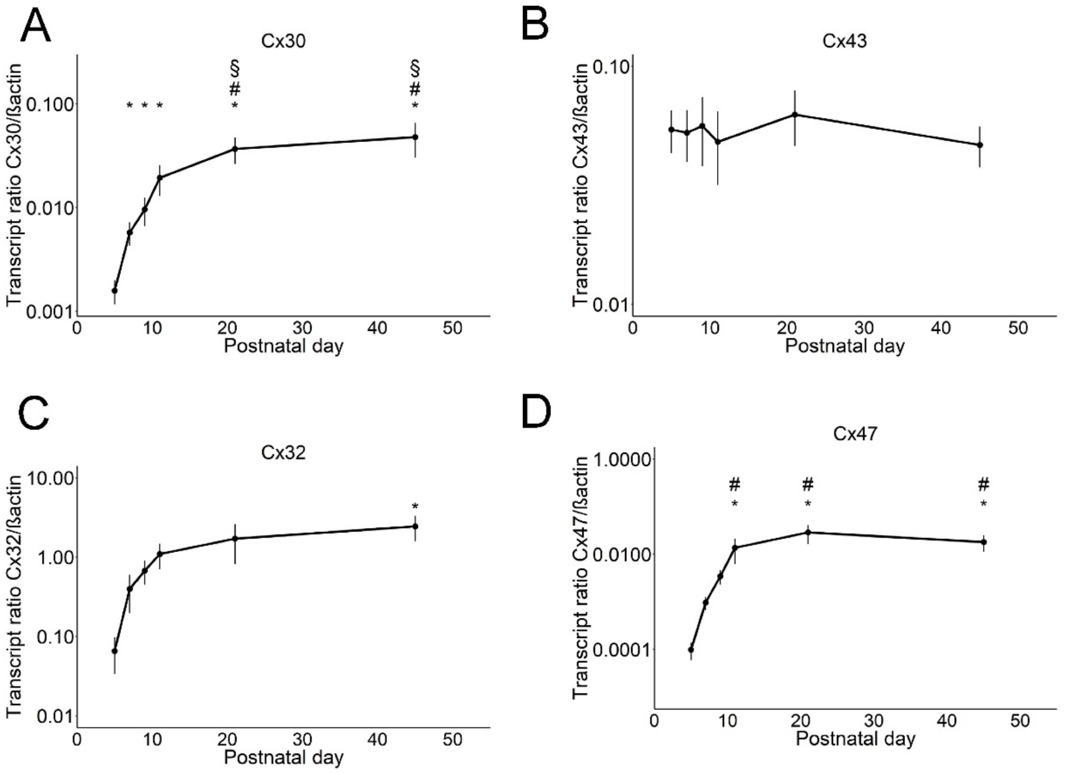

Suppl. Figure 1. Developmental regulation of Cx30, 43, 32 and 47 mRNA expression in the VPM. Gene expression ratios of Cx isoform/ß-actin according to equation (1) were plotted against postnatal age (p5, 7, 9, 11, 21, 45) (n = 4 to 9 samples per isoform). Data are depicted as mean ± standard error of the mean (SEM). Significant differences between p5 and other ages are indicated by (*), between p7 and older (#), and between p9 and older by (§). One way ANOVA and Tukey post-hoc test ($p < 0.05$) on log-transformed data or Kruskal-Wallis test followed by Dunn test. (A) Cx30: p5: 0.0016 ± 0.0004; p7: 0.0057 ± 0.0015; p9: 0.0096 ± 0.0029; p11: 0.0194 ± 0.0065; p21: 0.0368 ± 0.0104; p45: 0.0479 ± 0.0176. (B) Cx43: p5: 0.0543 ± 0.0110; p7: 0.0526 ± 0.0130; p9: 0.0562 ± 0.0180; p11: 0.0482 ± 0.0164; p21: 0.0627 ±0.0164; p45: 0.0467 ± 0.0091. (C) Cx32: p5: 0.0656 ± 0.0319; p7: 0.3983 ± 0.2021; p9: 0.6769 ± 0.2288; p11: 1.0953 ± 0.3913; p21: 1.7105 ± 0.8907; p45: 2.4444 ± 0.8633. (D) Cx47: p5: 9.88e-05 ± 3.96e-05; p7: 0.0010 ± 3.01e-04; p9: 0.0034 ± 1.16e-03; p11: 0.0137 ± 7.48e-03; p21: 0.0288 ± 1.23e-02; p45: 0.0181 ± 6.77e-03.

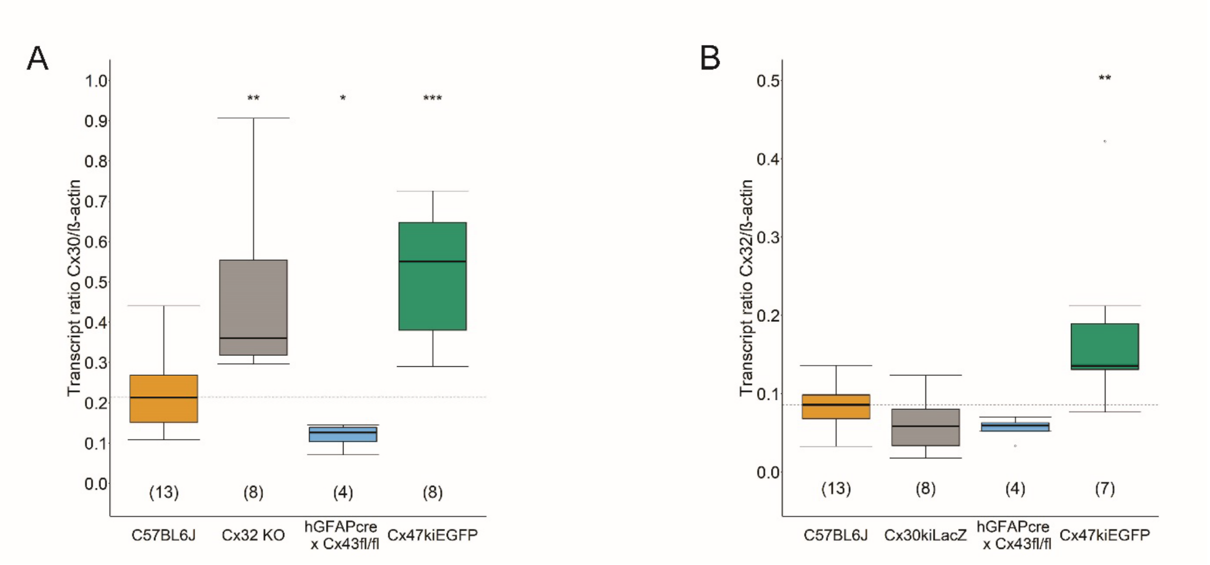

Suppl. Figure 2. (A) Altered Cx30 mRNA expression in the thalamus of Cx ko mice. Box plots compare Cx30 vs. □-actin transcript levels for VPM samples from control mice (0.2133, 0.1510 to 0.2687, n = 48, N = 13) with samples from Cx ko mice (Cx32 KO: 0.3603, 0.3181 to 0.5546, n = 24, N = 8, p = 0.002085; Cx47kiEGFP: 0.5511, 0.3801 to 0.6476, n = 25, N = 8, p = 0.0008823; hGFAPcre x Cx43fl/fl: 0.12623, 0.10379 to 0.13952, n = 12, N = 4, p = 0.04515). Dashed line indicates mRNA level in control samples. Asterisks indicate statistically significant differences (*p < 0.05, **p < 0.01, ***p < 0.001). Two sample t-test (hGFAPcre x Cx43fl/fl), Welch two sample t-test (Cx47kiEGFP) or Wilcoxon rank sum test (Cx32 KO). (B) Altered Cx32 mRNA expression in the thalamus of Cx ko mice. Box plots compare Cx32 vs. ß-actin transcript levels for VPM samples from control mice (0.08605, 0.06821 to 0.09851, n = 48, N = 13) with Cx ko mice (Cx30kiLacZ: 0.05852, 0.03390 to 0.08035, n = 24, N = 8; hGFAPcre x Cx43fl/fl: 0.05941, 0.05206 to 0.06297, n = 12, N = 4; Cx47kiEGFP: 0.13547, 0.13094 to 0.18927, n = 22, N = 7, p = 0.003541. Two sample t-test on log-transformed data). Box plots represent median and quartiles. Number of mice is given in brackets.

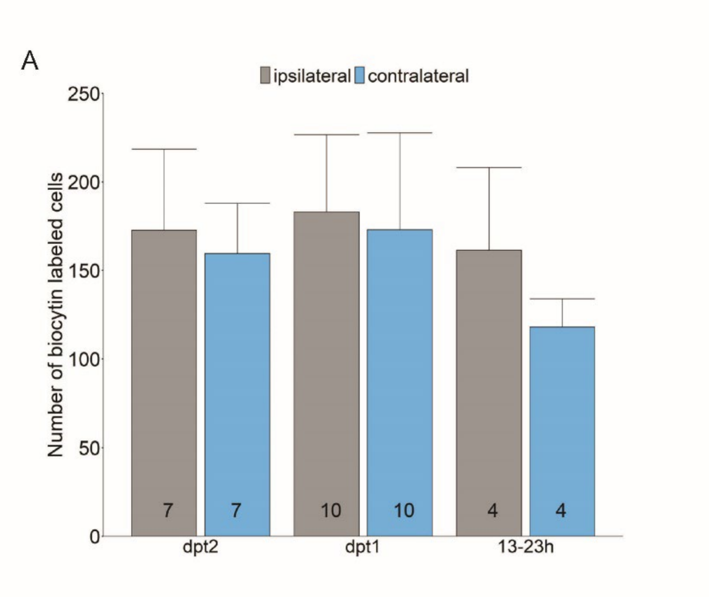

Suppl. Figure 3. Glial coupling efficiency in the VPM recovers at later time points after whisker trimming. Unilateral whisker trimming in C57BL6/J mice at p13-16 and analysis of tracer spread 2dpt, 1dpt or 13 – 23 h later did not reveal changes in coupling efficiency between the trimmed (contralateral) and control side (ipsilateral). Bar graphs represent mean ± SD. Number of mice is given in bar graphs. Two sample t-test.
